# Differential Expression of Mitosis and Cell Cycle Regulatory Genes during Recovery from an Acute Respiratory Virus Infection

**DOI:** 10.3390/pathogens10121625

**Published:** 2021-12-15

**Authors:** Ajinkya R. Limkar, Justin B. Lack, Albert C. Sek, Caroline M. Percopo, Kirk M. Druey, Helene F. Rosenberg

**Affiliations:** 1 Laboratory of Allergic Diseases, National Institute of Allergy and Infectious Diseases, National Institutes of Health, Bethesda, MD 20892, USA; limka001@umn.edu (A.R.L.); albertcsek@gmail.com (A.C.S.); percopoc@niaid.nih.gov (C.M.P.); kdruey@niaid.nih.gov (K.M.D.); 2 NIAID Collaborative Bioinformatics Resource, National Institute of Allergy and Infectious Diseases, National Institutes of Health, Bethesda, MD 20892, USA; justin.lack@nih.gov

**Keywords:** respiratory virus, pneumonia virus of mice, RNA sequencing, differential expression, mitosis, cell cycle regulation

## Abstract

Acute respiratory virus infections can have profound and long-term effects on lung function that persist even after the acute responses have fully resolved. In this study, we examined gene expression by RNA sequencing in the lung tissue of wild-type BALB/c mice that were recovering from a sublethal infection with the pneumonia virus of mice (PVM), a natural rodent pathogen of the same virus family and genus as the human respiratory syncytial virus. We compared these responses to gene expression in PVM-infected mice treated with *Lactobacillus plantarum*, an immunobiotic agent that limits inflammation and averts the negative clinical sequelae typically observed in response to acute infection with this pathogen. Our findings revealed prominent differential expression of inflammation-associated genes as well as numerous genes and gene families implicated in mitosis and cell-cycle regulation, including cyclins, cyclin-dependent kinases, cell division cycle genes, E2F transcription factors, kinesins, centromere proteins, and aurora kinases, among others. Of particular note was the differential expression of the cell division cycle gene *Cdc20b*, which was previously identified as critical for the ex vivo differentiation of multi-ciliated cells. Collectively, these findings provided us with substantial insight into post-viral repair processes and broadened our understanding of the mechanisms underlying *Lactobacillus*-mediated protection.

## 1. Introduction

Respiratory virus infections can have profound and long-term effects on lung function that persist even after the acute responses have fully resolved. Among other worries, the long-term physiologic sequelae of SARS-CoV-2 infection (i.e., long COVID) are currently of tremendous concern [1]. Even before the recent pandemic, numerous studies linked severe infection with respiratory syncytial virus (RSV) to the development of childhood wheezing and asthma [2,3]. The mechanisms underlying the connections between acute respiratory virus infection and long-term dysfunction remain unclear.

We and others have explored acute infection with the pneumonia virus of mice (PVM) as a model of severe human RSV infection (reviewed in [4,5,6,7]). PVM is a natural mouse pathogen of the same family (Pneumoviridae) and genus (Orthopneumovirus) as RSV. However, in contrast to RSV, PVM undergoes significant replication in mouse lung tissue and elicits a profound and frequently lethal inflammatory response [4,5,6]. In previous studies, we found that the administration of immunobiotic *Lactobacillus plantarum* directly to the respiratory tract had little to no impact on virus replication, but resulted in a profound reduction in the inflammatory response and full protection from the lethal sequelae of this infection [8,9,10,11,12].

We recently identified an inoculation strategy that resulted in symptomatic but sublethal PVM infection in wild-type mice [13]. Using this strategy, we found that all mice ultimately survived acute infection. This provided us for the first time with the opportunity to evaluate virus clearance, reversible weight loss, seroconversion, and airway hyperresponsiveness (AHR) during and after the recovery period, respectively.

In this study, we examined gene expression in the lung tissue of mice that are recovering from a sublethal PVM infection. We compared these responses to those of sublethal PVM-infected mice treated with *L. plantarum* and those treated with *L. plantarum* alone. Our findings revealed the prominent differential expression of inflammation-associated genes as well as numerous genes and gene families implicated in mitosis and cell-cycle regulation. Collectively, these findings provide us with substantial insight into post-viral repair processes and broaden our understanding of the mechanisms underlying *Lactobacillus*-mediated protection.

## 2. Methods

### 2.1. Mice

Wild-type female BALB/c mice (6–10 weeks old) were purchased from Charles River Laboratories. Mice were maintained on-site at the National Institutes of Allergy and Infectious Diseases (NIAID) of the National Institutes of Health. All mice were maintained under pathogen-free conditions at an American Association for the Accreditation of Laboratory Animal Care accredited animal facility at the NIAID and housed in a 14BS vivarium in accordance with the procedures outlined in the Guide for the Care and Use of Laboratory Animals under an animal study proposal approved by the NIAID Animal Care and Use Committee. In vivo efficacy studies were approved by the NIAID Institutional Animal Care and Use Committee. Animal work was conducted adhering to the institution’s guidelines for animal use and followed the guidelines and basic principles in the United States Public Health Service Policy on Humane Care and Use of Laboratory Animals, and the Guide for the Care and Use of Laboratory Animals by certified staff in an Association for Assessment and Accreditation of Laboratory Animal Care (AAALAC) International accredited facility as per protocol LAD-8E.

### 2.2. Inoculation with PVM and Immunobiotic L. plantarum

**Figure 1:**
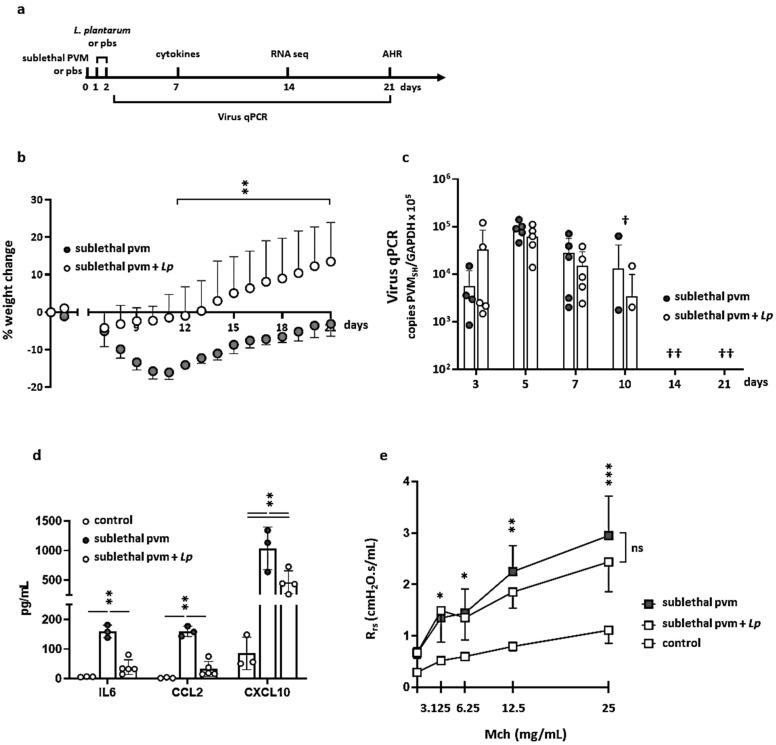
The administration of *L. plantarum* to the respiratory tract prevents weight loss and inflammation in response to a sublethal inoculum of PVM. (**a**) Experimental timeline. Mice were intranasally inoculated with a sublethal dose of PVM (27 virus copies in 50 μL PBS) or PBS control on day 0 followed by *L. plantarum* (*Lp*; 10^8^ cells in 50 μL PBS) or PBS control on days 1 and 2. Weight loss, virus replication (qRT-PCR), inflammation, and total airway resistance (AHR) were evaluated on the days indicated. (**b**) Percent original weight (± SD) exhibited by mice inoculated with sublethal PVM alone or sublethal PVM +*Lp*; n = 4–5 mice per group, * *p* < 0.05; ** *p* < 0.01 (2-way ANOVA with Sidak’s multiple comparison test). (**c**) Virus detected in lung tissue by qRT-PCR on days 3, 5, 7, 14, and 21 as indicated in (**a**); n = 5 mice per group, ^†^ virus undetectable in 3 of 5 mice; ^††^ virus undetectable in all mice. (**d**) Immunoreactive IL-6, CCL2, and CXCL10 detected in BAL.fluid on day 7 in response to sublethal PVM infection alone or sublethal PVM +*Lp* as indicated in (**a**); n = 3–5 mice per group, ** *p* < 0.01 (2-way ANOVA with Tukey’s multiple comparisons test). (**e**) Airway resistance (R_rs_; cmH_2_O.s/mL) in response to increasing concentrations of methacholine (Mch; 0–25 mg/mL) evaluated on day 21 after inoculation with a sublethal dose of PVM alone or sublethal PVM +*Lp*; n = 3–5 mice per group; * *p* < 0.05 vs. control; ** *p* < 0.01 vs. control; *** *p* < 0.001 vs. control; ns, not significant.

The pneumonia virus of mice (PVM) strain J3666 was prepared from clarified mouse-passaged stocks stored in liquid nitrogen as previously described [8]. Mice under isoflurane anesthesia were intranasally inoculated on day 0 with 50 µL PVM diluted to 27 virus copies/mL (as determined by qPCR [14]) in phosphate-buffered saline (PBS) with 0.1% bovine serum albumin (BSA) or diluent control (PBS/BSA). *L. plantarum* NCIMB 8826 (BAA-793; ATCC) was grown overnight in DeMan Rogosa Sharp medium, heat-inactivated (70 °C for 30 min), washed once in sterile PBS and stored at 10^11^/mL at −20 °C in sterile PBS with 0.1% BSA. The conversion from OD to cells/mL was originally described in reference [8]. *L. plantarum* (10^8^ cells in 50 µL PBS/BSA or PBS/BSA alone) was administered to the respiratory tract on days 1 and 2 as indicated in Figure 1a. Weights were assessed daily through day 21 post-inoculation. Other mice were sacrificed for evaluation at the time points indicated. Bronchoalveolar lavage (BAL) fluid was collected via two washes, each with 0.8 mL PBS/BSA. BAL fluid was clarified by centrifugation and stored at −80 °C prior to analysis.

### 2.3. Enzyme-Linked Immunosorbent Assays (ELISAs)

Proinflammatory cytokines detected on day 7 after inoculation with a sublethal dose of PVM alone (on day 0), sublethal PVM +*Lp* (on days 1 and 2), or diluent alone were evaluated by ELISA (DuoSet, R&D Systems).

### 2.4. Measurements of Airway Resistance

Airway resistance (R_rs_) was assessed as previously described [13]. Briefly, mice (day 21 after inoculation as per Figure 1a) were anesthetized by the intraperitoneal injection of ketamine and xylazine before undergoing tracheal intubation. The mice were mechanically ventilated at a rate of 150 breaths/min, with a tidal volume of 10 mL/kg and positive-end expiratory pressure of 3 cm H_2_O. Before the start of ventilation, mice were paralyzed by intraperitoneal injection of vecuronium bromide solution (0.1 mL of a 0.1 mg/mL solution). During data collection, increasing doses of methacholine (Mch; 0, 3.125, 6.25, 12.5, and 25 mg/mL) in PBS were directly nebulized into the lungs of mice. Airway resistance was measured using the Scireq flexiVent system (Montreal, WI, USA). Data are presented as means ± standard deviation (SD) of maximum resistance values (R_rs_) in units of cmH_2_O/mL/s.

### 2.5. RNA Extraction from Whole Lung Tissue

Lungs were removed from the body cavity, washed, and immediately homogenized in 3 mL RNA Bee reagent (AMSBIO) followed by chloroform extraction and precipitation with 70% ethanol. RNA was isolated and purified using an RNeasy Mini or RNeasy MinElute cleanup kit (Qiagen). Purified RNA was eluted in RNase-free water and quantified using a Nanodrop One. RNA integrity (RIN) was evaluated on an Agilent Bioanalyzer. All samples used in the RNA sequencing experiments exhibited RIN values > 8.0.

### 2.6. RNA Sequencing

RNA libraries were prepared using the Illumina TruSeq Stranded mRNA Library Prep Kit following the manufacturer’s instructions. Paired end-reads of the RNA libraries were sequenced using an Illumina NextSeq 500 which generated >35 million reads per sample. Raw fastq files were then trimmed for quality and adapter contamination using Cutadapt v2.10 [15] and trimmed reads were mapped to the mm10 mouse reference genome and Gencode M25 transcriptome using STAR v2.5.3 [16] in two-pass mode. Gene-level expression quantification was performed using RSEM v1.3.0 [17] and standard differential expression was performed using the R package limma with voom normalization [18]. Prior to differential expression and downstream timecourse analysis, genes were filtered that had <1 counts per million (CPM) across <3 samples. Gene networks were generated using IDEP (http://bioinformatics.sdstate.edu/idep93/accessed on 13 December 2021) which uses the visNetwork R package (https://datastorm-open.github.io/visNetwork/ (accessed on 13 December 2021)) to collapse enrichment pathways into clusters. Connections were drawn between pathways that share ≥20% of their associated genes; edge thickness is proportional to the percent overlap in gene content between two connected nodes. Principal component analysis revealed appropriate clustering (Supplemental Figure S1) save for the one sample indicated by the arrow. This sample was omitted from further analysis.

### 2.7. Statistical Analysis

Statistical analyses for datasets other than those generated by RNA sequencing were performed using algorithms in GraphPad Prism 9.2.0 as indicated in the figure legends.

## 3. Results

### 3.1. Impact of L. plantarum in Mice with Sublethal PVM Infection

As noted above, we recently identified an inoculation strategy that generates a symptomatic but sublethal PVM infection in wild-type mice. Outcomes from this strategy include reversible weight loss accompanied by virus replication and ultimately seroconversion [13]. In earlier studies, we found that mice inoculated with the standard (lethal) dose of PVM were protected from the sequelae of severe infection by the direct administration of *L. plantarum* to the airways either before or immediately after inoculation [8,9,10,11,12]. Here, we examined the impact of the administration of heat-inactivated *L. plantarum* on weight loss, cytokine production, virus replication, airway resistance, and gene expression in mice with a sublethal PVM infection (Figure 1a).

As shown in Figure 1b, BALB/c mice begin to lose weight on day 7 after inoculation with a sublethal dose of PVM. Maximum weight loss (−15.9 ± 1.9% of original body weight) was observed on days 10–11. Average weights returned to baseline levels by day 21. PVM-infected mice that were treated with *L. plantarum* on days 1 and 2 exhibited weights that were at or above baseline throughout and maintained weights that were significantly higher than those exhibited by mice inoculated with the sublethal dose of PVM alone from days 10–21.

Similar to our findings in mice with lethal PVM infection, the administration of *L. plantarum* had no impact on virus replication or clearance. As shown in Figure 1c, viral RNA was detected in fewer than half the mice (both those infected with PVM alone and those also treated with *L. plantarum*) examined on day 10; no virus was detected in any mice on days 14 and 21. By contrast, the administration of *L. plantarum* suppressed the virus-induced inflammatory response. Similar to our findings from the lethal infection model, treatment with *L. plantarum* resulted in significant reductions in the levels of immunoreactive IL-6, CCL2, and CXCL10 detected in the BAL fluid of mice inoculated with a sublethal dose of PVM (Figure 1d).

The development of a symptomatic but sublethal PVM model provided us with the opportunity to examine changes in the airway that persist after virus clearance and recovery. In our previous study [13], we found that mice that recovered from a sublethal dose of PVM developed significant airway hyperresponsiveness that could be detected as early as day 21 post-inoculation. Interestingly, and despite its impact on both weight loss and proinflammatory cytokine production, treatment with *L. plantarum* resulted in no significant reductions in total methacholine-induced airway resistance (R_rs_) measured at this time point (Figure 1e).

### 3.2. Gene Expression during Recovery from an Acute Sublethal PVM Infection

We previously reported the results of a microarray study featuring total lung tissue from mice on day 5 after inoculation with a lethal dose of PVM that revealed profound differential expression of numerous proinflammatory cytokine genes [9]. Here, we performed RNA sequencing on total lung tissue from mice on day 14 after inoculation with a sublethal dose of PVM or diluent control either with or without *L. plantarum* as shown in Figure 1a. Our goal was to identify genes, networks, and pathways that were differentially regulated during recovery from acute infection. All PVM-infected mice cleared the virus from lung tissue at this time point (see Figure 1c). Mice inoculated with PVM alone exhibit significant recovery from peak weight loss at this time point (−11.0 ± 1.7% on day 14 vs. −15.9 ± 1.9% on days 10–11, *p* < 0.001; see Figure 1b).

The four groups of mice evaluated included: (a) mice infected with a sublethal dose of PVM on day 0 and diluent control on days 1 and 2 (+pvm); (b) mice infected with a sublethal dose of PVM on day 0 and treated with *L. plantarum* on days 1 and 2 (+pvm +*Lp*); (c) mice inoculated with diluent control on day 0 and treated with *L. plantarum* on days 1 and 2 (+*Lp*); and (d) mice inoculated with diluent control on days 0, 1, and 2 (control). Quantitative comparisons between these groups are shown in Table 1. We identified 289 genes that were up-regulated (log2|FC| ≥ 2.00, or ≥ 4-fold) and 19 that were down-regulated (log_2_|FC| ≤ −2.00) in response to PVM alone (+pvm vs. control) at this time point. In contrast, only 86 genes were upregulated ≥ 4-fold in the +pvm *+Lp* group vs. control. Interestingly, we also identified 68 genes that were upregulated and 8 genes that were down-regulated in response to inoculation with *L. plantarum* alone (+*Lp* vs. control).

### 3.3. Differential Expression of Proinflammatory Genes

**Figure 2:**
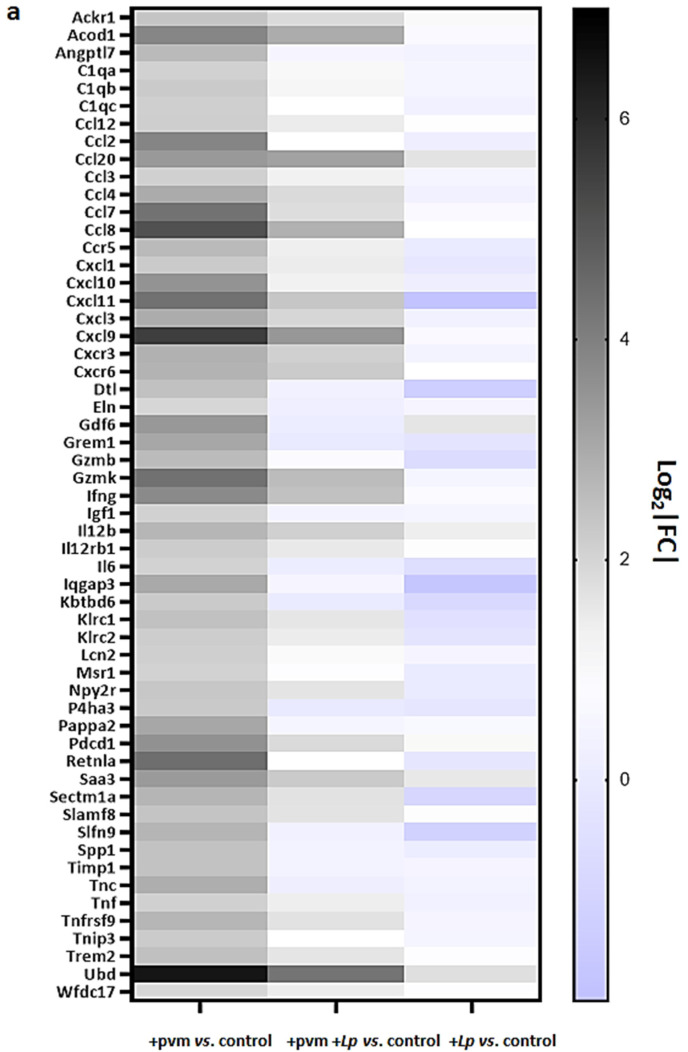

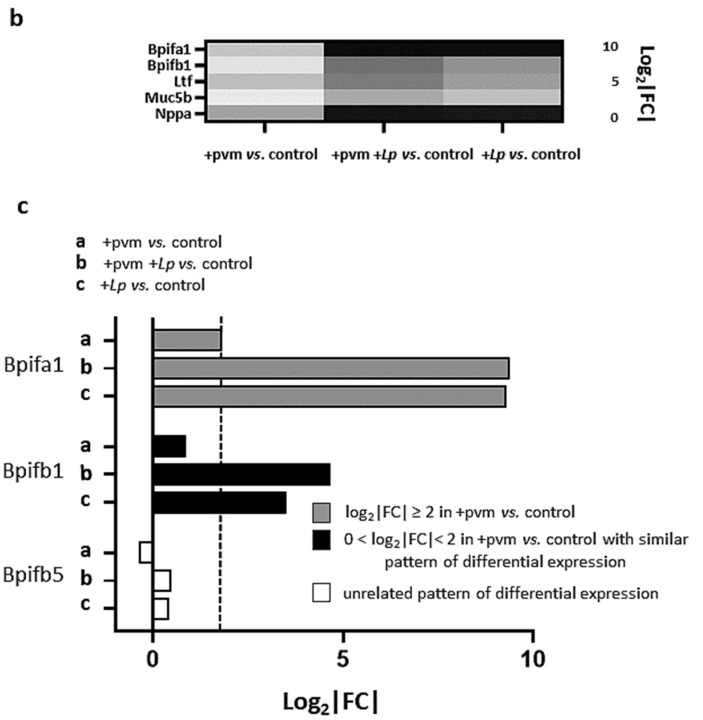
**Differential expression of inflammation-associated genes in mouse lung tissue.** (**a**) Inflammation-associated genes upregulated ≥ 4-fold or more (log_2_|FC| ≥ 2) during recovery from acute sublethal PVM infection vs. control were identified in mouse lung tissue by RNA sequencing (n = 4 mice per group). These responses were compared to those exhibited by mice inoculated with PVM +*Lp* vs. control or *Lp* alone vs. control (n = 3–4 mice per group). (**b**) Inflammation-associated genes upregulated ≥ 4-fold or more (log_2_|FC| ≥ 2) in response to the administration of *Lp* alone vs. control identified in mouse lung tissue by RNA sequencing (n = 4 mice per group). These responses were compared to those exhibited by mice inoculated with PVM +*Lp* vs. control or PVM vs. control alone (n = 3–4 mice per group). (**c**) Expression patterns of differentially expressed genes encoding members of the *Bpif* family; grey-filled bars, genes upregulated 11–600-fold in response to *Lp*; white-filled bars, genes responding minimally under these conditions. Ensembl IDs, gene names, functional annotation, and fold-increases for each entry are included in Supplementary Table S1 and/or GSE186740.

As anticipated from previous studies [8,9,10,11,12], the RNA sequencing data revealed profound differential expression of numerous genes involved in the inflammatory response. The results presented in Figure 2a document the differential expression of 55 genes associated with pro-inflammatory pathways, including *Il6* (4-fold up-regulation in +pvm vs. control), a cytokine with profound adverse impact on the outcome of lethal PVM infection [11]. In contrast, we detected minimal (1.04-fold) up-regulation and 1.5-fold down-regulation of *Il6* when comparing +pvm +*Lp* vs. control and +*Lp* alone vs. control, respectively. Other transcripts encoding pro-inflammatory genes identified as up-regulated in response to sublethal PVM infection and down-regulated in response to *L. plantarum* include those encoding *Ccl2*, *Ccl8*, *Cxcl9*, *Cxcl10*, and resistin-like molecule alpha (*Retnla*). While Retn1a (also known as FIZZ-1) has been identified as a critical regulator of allergic inflammation [19,20], Samarasinghe and colleagues [21] reported that the direct administration of recombinant Retnla and Retnlb to the airways resulted in a significant reduction in host immune responses in mice infected with influenza A.

Several differentially regulated genes associated with the inflammatory response have also been implicated in tissue remodeling, including those encoding elastin (*Eln*) and tissue inhibitor of matrix metalloproteinase 1 (*Timp1*). Similar to the cytokines described above, these mediators have also been primarily characterized in mouse models of allergic airways and tissue fibrosis [22,23,24,25]. Our understanding of their role in the post-virus recovery period remains to be explored.

The experimental design of this study also provided us with the opportunity to examine gene expression in response to administration of *L. plantarum* alone and to obtain additional insight into its protective mechanism. Of the 68 genes that were up-regulated in response to *L. plantarum* alone, we identified five that were associated with pro-inflammatory pathways (Figure 2b). These include genes encoding natriuretic peptide A (*Nppa*), a cardiac hormone that regulates fluid balance and limits endothelial dysfunction and vascular leakage in the lung in response to acute inflammatory injury [26], and *Muc5b*, which encodes a major mucin of the respiratory tract that has been associated with mucociliary dysfunction [27,28].

*Bpifa1* and *Bpifb1* were also up-regulated (600- and 11-fold, respectively) in response to *L. plantarum*; these mediators are specifically expressed in respiratory epithelial cells and encode proteins that interact with bacteria [29]. We observed a minimal differential regulation of *Bpifb5* (Figure 2c) and no differential regulation of any of the other members of this specific gene family. Interestingly, Akram and colleagues [30] found that endogenous Bpifa1 (and also Muc5b) served to limit the replication of influenza A/H3N2 in a tracheal air–liquid interface cell culture model. A future study might address the potential roles of Bpifa1 and Bpifb1 in limiting the inflammatory sequelae of PVM infection.

### 3.4. Differential Expression of Genes Associated with Mitosis and Cell Cycle Regulation

**Figure 3:**
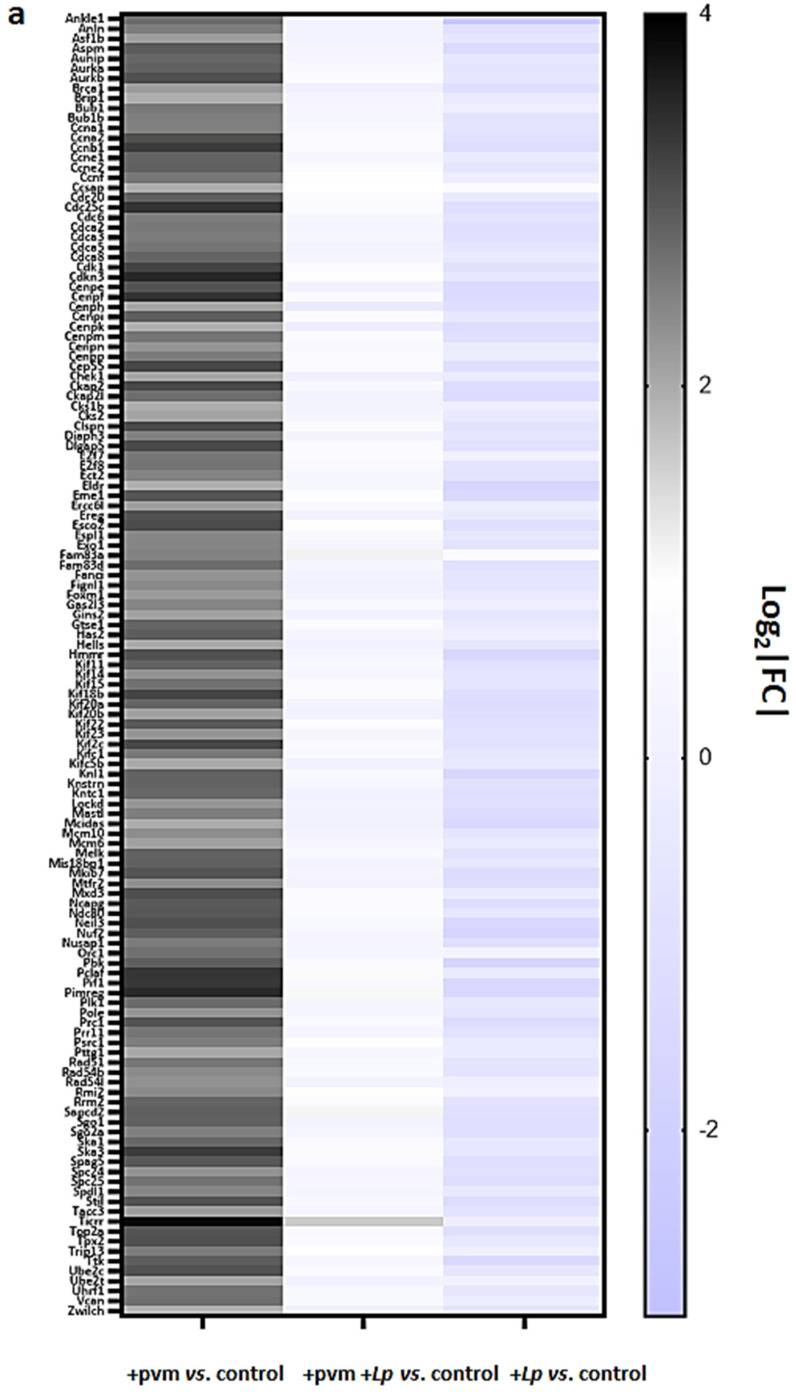

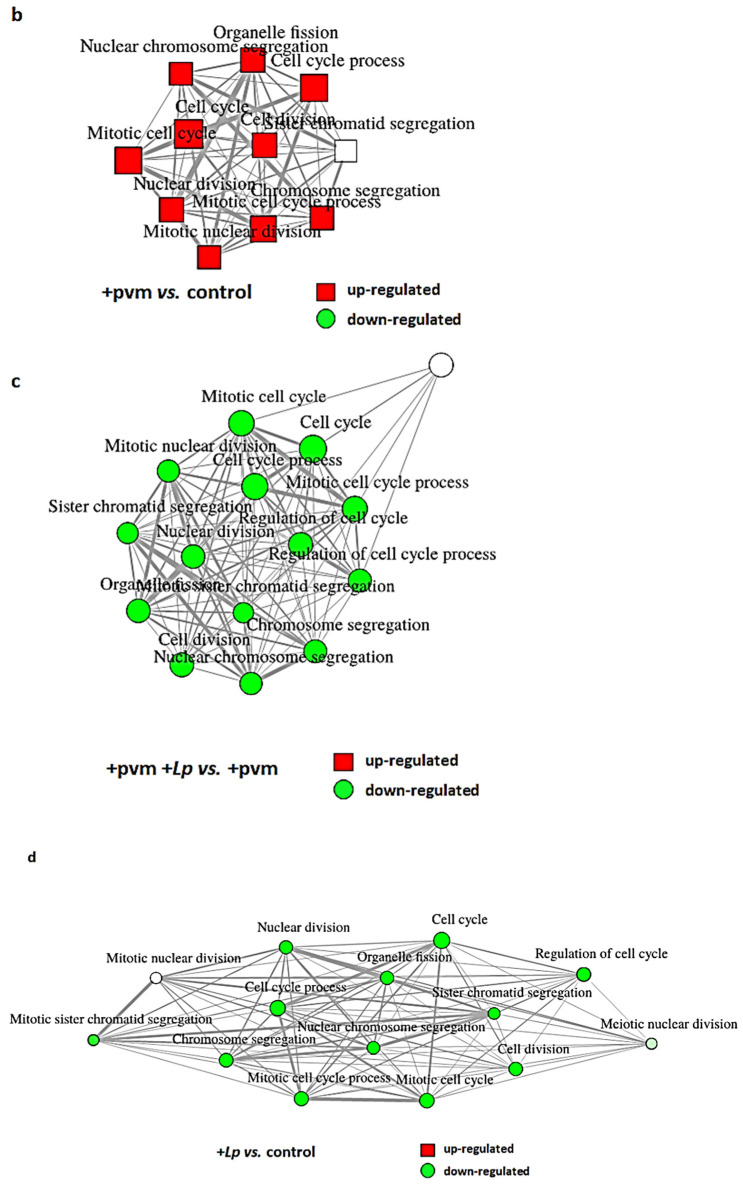
**Differential expression of mitosis and cell cycle regulation genes in mouse lung tissue.** (**a**) Mitosis and cell cycle regulatory genes up-regulated ≥ 4-fold or more (log_2_|FC| ≥ 2) during recovery from an acute sublethal PVM infection (+pvm vs. control) identified in mouse lung tissue by RNA sequencing (n = 4 mice per group). These responses were compared to those exhibited by mice inoculated with PVM +*Lp* vs. control or *Lp* alone vs. control (n = 3–4 mice per group). Ensembl IDs, gene names, functional annotation, and fold-increases for each entry are included in Supplementary Table S2 and GSE186740. (**b**–**d**) Gene network diagrams highlighting differential expressions and functional links between mitosis and cell cycle regulatory genes in response to PVM vs. control, PVM +*Lp* vs. PVM, and *Lp* vs. control, respectively.

Successful recovery from an acute respiratory virus infection involves wide-ranging and dramatic changes throughout the lung tissue, most notably events and responses that support the repair and reconstruction of the respiratory epithelium [31,32]. As discussed in the first section above, day 14 of our protocol represents an early point in this recovery process; the virus was cleared from the lung tissue, and mice infected with PVM alone were gaining weight at a rate of ~1.7 g/day to recover homeostasis (see Figure 1b). Interestingly, we found that 131 of the 289 (45%) of the genes identified as up-regulated in response to PVM infection encode proteins that are directly associated with cell cycle regulation and mitosis (Figure 3a,b). Without exception, the expression of each of these 131 transcripts was diminished when comparing the responses of PVM-infected mice treated with *L. plantarum* (+pvm +*Lp* vs. control) to those of PVM-infected mice alone (+pvm vs. control; Figure 3c). Of particular interest, nearly all of these transcripts (128 of 131, or 98%) were down-regulated from baseline control levels in mice treated with *L. plantarum* alone (Figure 3d). The significance of this observation will be further considered in the Discussion.

Gene families that are highly represented in this group include the cyclins (CCNs), the cyclin-dependent kinases (CDKs) and their associated proteins, the E2F family of transcription factors (E2Fs), cell division cycle genes (CDCs), kinesins (KIFs), centromere proteins (CENPs), and Aurora kinases (AURKs) and interacting proteins.

Collectively, these findings reveal a dramatic induction of genes and pathways associated with mitosis and cell cycle regulation during recovery from an acute sublethal PVM infection and their corresponding diminished expression in response to treatment with *L. plantarum*. However, it is critical to recognize that the results shown here represent a virtual snapshot of differential gene expression taken at a single point in time. Likewise, the study was performed using total lung tissue which by its nature includes a large variety of cell types (e.g., epithelial cells, endothelial cells, airway smooth muscle cells, and leukocytes) all interacting with one another within the larger environment of the lung. While one cannot generate a full interpretation of these findings based on the contributions of the multitude of asynchronized cells, they serve as a starting point for future consideration and the experimental exploration of the role(s) of specific proteins and pathways in critical in vivo settings. The reader is referred to several recent reviews for additional insight into the molecular basis of cell cycle regulation [33,34,35].

### 3.5. Differential Expression of Genes Encoding Cyclins (CCNs), Cyclin-Dependent Kinases (CDKs), Cell-Division Cycle Genes (CDCs), and E2F Transcription Factors

**Figure 4:**
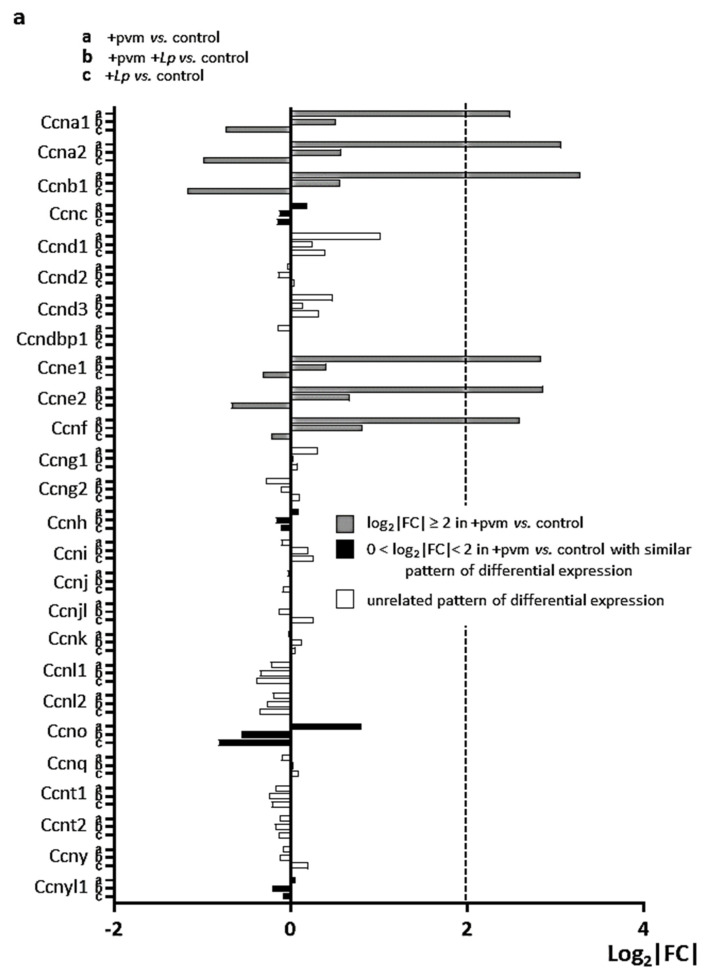

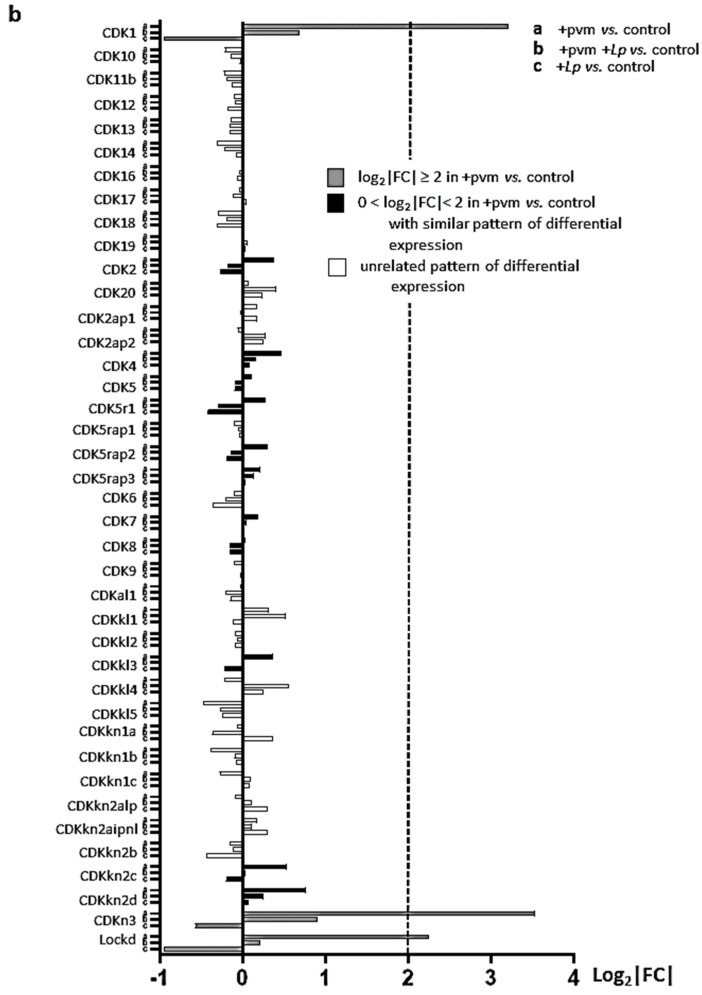

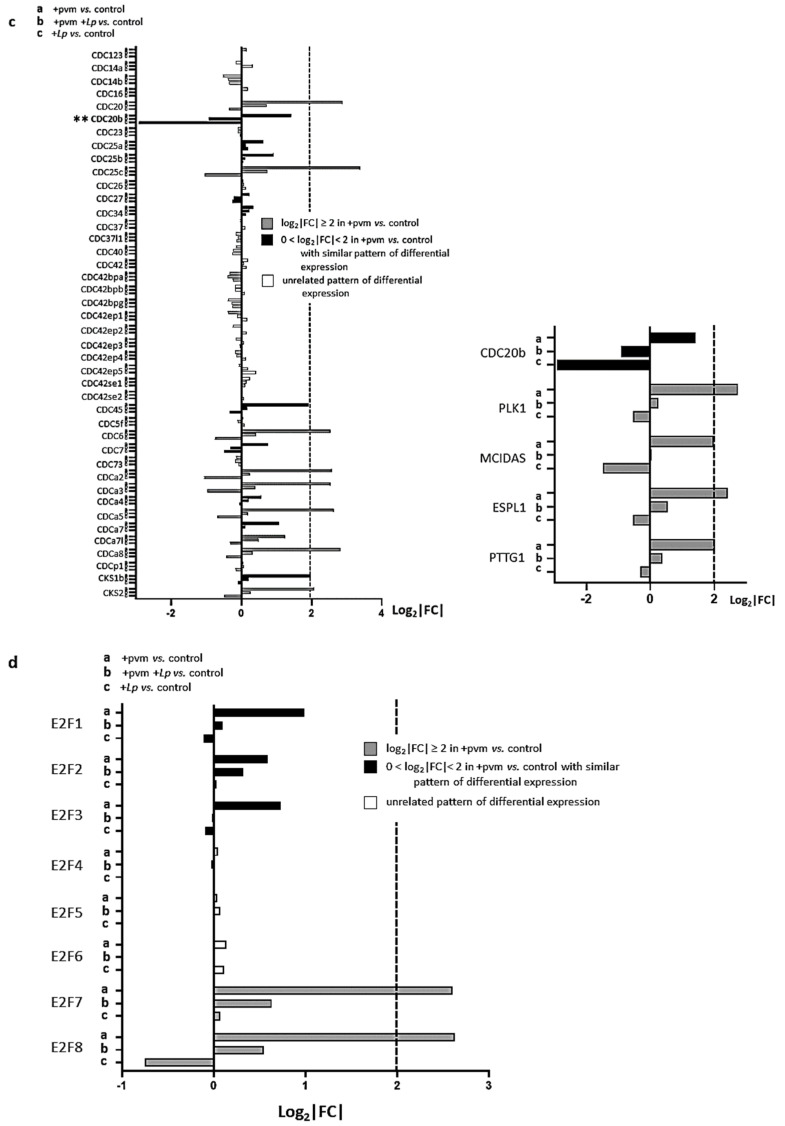
Differential expression of mitosis and cell cycle regulation gene families during recovery from an acute PVM infection. Expression of (**a**) cyclins (*Ccn*s), (**b**) cyclin-dependent kinases (*Cdk*s), (**c**) cell division cycle proteins (*Cdc*s), and (**d**) E2F transcription factors in PVM-infected mice (+pvm), PVM-infected mice treated with *L. plantarum* (+pvm +*Lp*) and mice treated with *L. plantarum* alone (+*Lp*). The inset in (**c**) highlights the differential expression of *Cdc20b* and includes additional transcripts that have been associated with centriole formation in ciliated airway epithelial cells. Grey-filled bars represent transcripts that were up-regulated 4-fold or more (log_2_|FC| ≥ 2) in the +pvm vs. control group; black-filled bars represent transcripts that were up-regulated in the +pvm vs. control group and followed a similar differential expression pattern, although they did not reach the 4-fold threshold; white bars represent transcripts with an unrelated pattern of differential expression. Ensembl IDs, gene names, functional annotation, and fold-increases are listed in Supplementary Table S2 and GSE186740.

***Cyclins:*** Cyclins (CCNs) are a collection of complex proteins (MW 30–90 kDa) that regulate cell growth and division and promote progression through the cell cycle via their regulatory interactions with specific CDKs [36,37]. Differential expression of 26 CCN genes during recovery from an acute PVM infection with or without *L. plantarum* treatment is shown in Figure 4a. PVM infection alone results in a profound upregulation of select CCN genes, including *Ccna1*, *Ccna2*, *Ccnb1*, *Cce1*, *Ccne2*, and *Ccnf*; these responses are diminished in PVM-infected mice treated with *L. plantarum*. As noted above, gene expression levels fall below levels detected in controls in mice treated with *L. plantarum* alone. Among these differentially expressed CCN genes, *Ccna1* encodes an alternative A-type cyclin that interacts with Cdk2 and E2F-1 [38] and promotes cell proliferation via G1 to S progression through the cell cycle [39]. In contrast, Ccnb1 interacts with Cdk1 and promotes the synchronization of the cell cycle with mitochondrial bioenergetics [40]. Ccnf resembles Ccna (i.e., similar sequence and expression patterns [41]), however, it can regulate the cell cycle without partnering with a CDK [42].

***Cyclin-dependent kinases*:** Cyclin-dependent kinase (*Cdk*) genes encode several evolutionarily divergent subfamilies of proteins that interact with specific CCNs to regulate progression through the cell cycle [43,44,45,46]. The differential expression of 40 *Cdk* genes is shown in Figure 4b. Interestingly, PVM-infection alone results in the substantial up-regulation of only one CDK gene (*Cdk1*) and one CDK inhibitor (*Cdkn3*), and only minimal differential regulation of *Cdk4*, *Cdk6*, or *Cdk8*, which are genes that encode the specific partners of the aforementioned differentially regulated CCN genes. However, these results are consistent with findings reported in a review by Arellano and Moreno [47] who noted that, unlike CCNs, CDK levels remain relatively constant throughout the cell cycle. Nonetheless, the expression of both *Cdk1* and *Cdkn3* were suppressed in PVM-infected mice treated with *L. plantarum*, and similar to the pattern described for the CCNs (see Figure 4a), expression levels of these genes fall below baseline in mice treated with *L. plantarum* alone. Of note, *Cdk1* encodes a 34 kDa protein that interacts with CCNa and CCNb to promote the transition from S to G2 and G2 to M phases, respectively, and is the only CDK that is absolutely required for mammalian cell cycling [48].

***Cell Division Cycle Genes*:** Cell division cycle (CDC) genes were first described by Hartwell, Nurse, and colleagues [49,50] in functional studies carried out in *Saccharomyces cerevisiae*. Some (but not all) of these genes have since been identified as encoding CCNs and CDKs. Numerous CDC genes are differentially expressed in the lung tissue of mice recovering from an acute PVM infection, including *Cdc20*, *Cdc20b*, *Cdca3*, *Cdca4*, and *Cdca7* (Figure 4c).

We were interested to compare our findings to those of Revinski and colleagues [51] who reported that the expression of the vertebrate-specific gene encoding CDC20b was critical for the generation and differentiation of multi-ciliated cells, including mouse tracheal epithelial cells in air–liquid interface cultures. Furthermore, their results revealed the co-expression of *CDC20b* with the mitotic kinase, *PLK1,* and *MCIDAS* (Multiciliate Differentiation Additionally, DNA Synthesis Associated Cell Cycle Protein) during centriole formation in human airway epithelial cells. As shown in the inset to Figure 4c, both *Plk1* and *Mcidas* were up-regulated in lung tissue of mice recovering from an acute PVM infection as were the PLK1-interacting partner, Separase (*Espl1*), and its inhibitor, Securin (*Pttg1*) which are both critical for promoting centriole duplication [52,53]. Interestingly, we detected no significant differential expression of lung-specific transcripts *Aqp5* [54] or any of the surfactants [55,56,57] at the day 14-time point. Nonetheless, these results suggest that the upregulation of *Cd20b* and the aforementioned associated genes may substantially contribute to the repair of damaged lung tissue during recovery from acute respiratory virus infection.

As we noted earlier in the general discussion of the differential expression patterns, our findings revealed that the expression of *Cdc20b* and associated transcripts were all down-regulated in PVM-infected mice treated with *L. plantarum* (+pvm +*Lp* vs. control) compared to the responses of pvm-infected mice alone (+pvm vs. control). Likewise, all of these genes were detected at levels that were below baseline controls in mice treated with *L. plantarum* alone. Interestingly, *Cdc20b* exhibited the largest response (7.5-fold) of the eight genes identified as down-regulated in response to *L. plantarum* alone. This observation will be further considered in the Discussion.

***E2F transcription factors*:** The E2F transcription factors are the master regulators of the mammalian cell cycle that activate or repress the expression of critical cell cycle genes via their interactions with retinoblastoma susceptibility protein (pRB) and CDKs. Eight distinct E2F factors were identified in mammals that directly bind to a single consensus binding sequence (reviewed in [58,59]). Our results reveal differential expression of numerous E2F genes, most notably genes encoding the atypical repressors E2F7 and E2F8 (Figure 4d). While E2Fs target a vast array of cell signaling proteins, E2F7 and E2F8 are induced by and mediate transcriptional repression in response to DNA damage via interactions with the promoters of genes encoding E2F1, E2F2, and E2F3a [60,61].

### 3.6. Differential Expression of Genes Encoding Kinesins (KIFs), Centromere Proteins (CENPs), and Aurora Kinases

**Figure 5:**
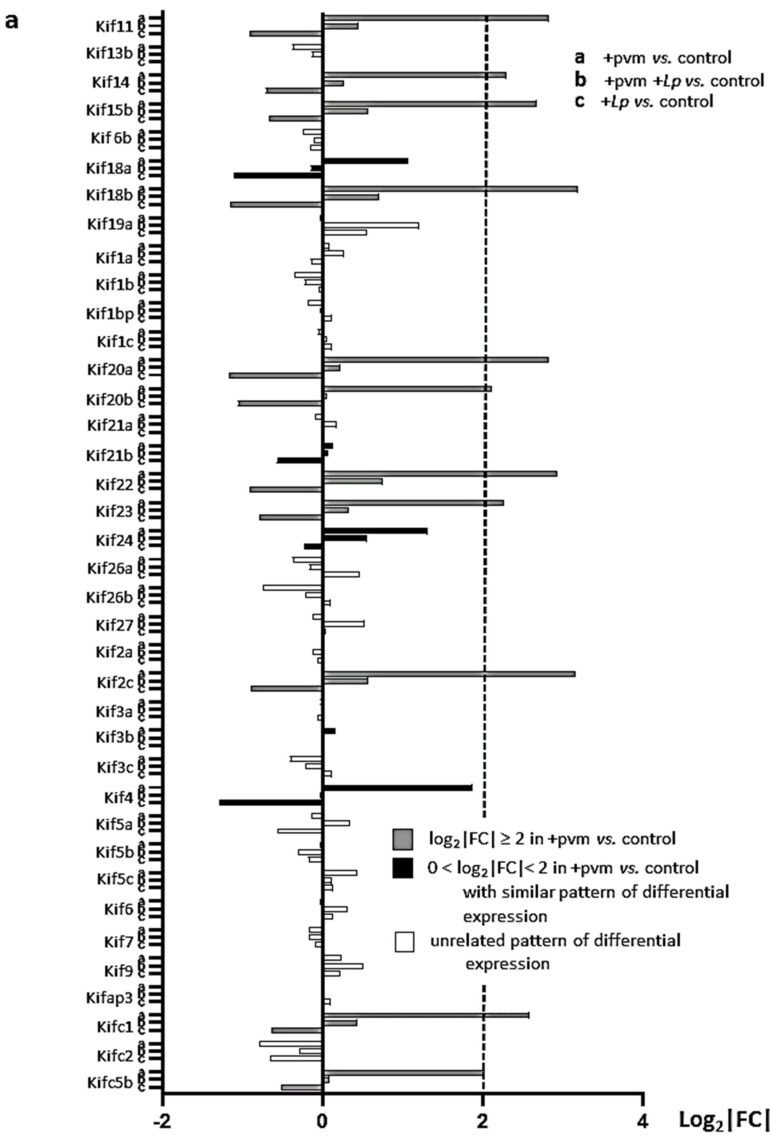

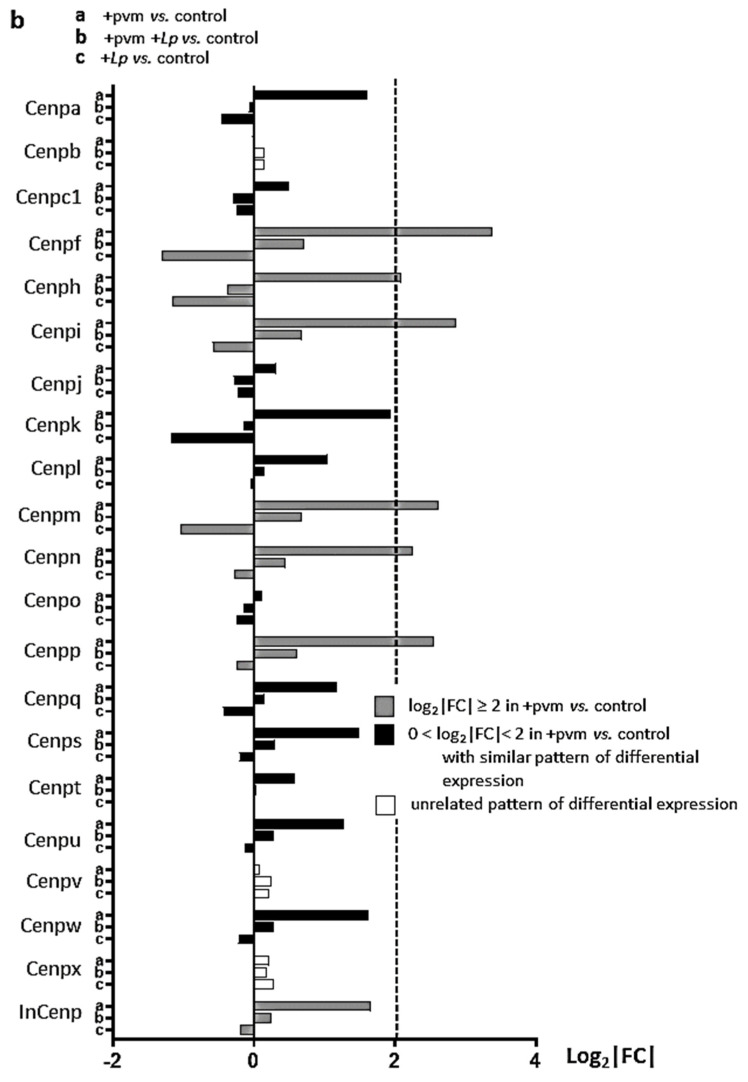

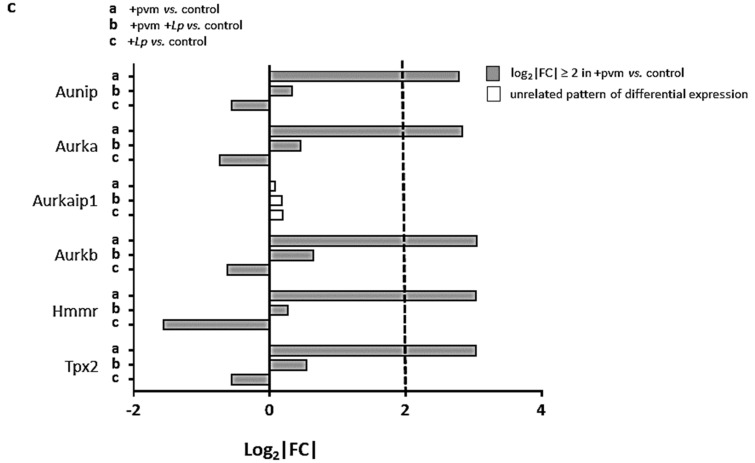
Differential expression of additional mitosis and cell cycle regulation gene families during recovery from an acute PVM infection. Expression of (**a**) kinesins (*Kif*s), (**b**) centromere proteins (*Cenp*s), and (**c**) aurora kinases (*Aurk*s) and their interacting partners in PVM-infected mice (+pvm), PVM-infected mice treated with *L. plantarum* (+pvm +*Lp*), and mice treated with *L. plantarum* alone (+*Lp*). Grey-filled bars represent transcripts that were up-regulated 4-fold or more (log_2_|FC| ≥ 2) in the +pvm vs. control group; black-filled bars represent transcripts that were up-regulated in the +pvm vs. control group and followed a similar differential expression pattern, although they did not reach the 4-fold threshold; white bars represent transcripts with an unrelated pattern of differential expression. Ensembl IDs, gene names, functional annotation, and fold-increases are listed in Supplementary Table S2 and GSE186740.

***Kinesins*:** Kinesins are ATPases encoded by *Kif* genes that modulate microtubule activity in support of numerous cell functions, including mitosis [62,63,64]. Our findings reveal profound up-regulation of genes encoding various kinesins in lung tissue of mice recovering from a sublethal PVM infection, as well as their diminished expression in PVM-infected mice treated with *L. plantarum* or mice treated with *L. plantarum* alone (Figure 5a). Among these are genes encoding kinesin-4 (encoded by *Kif4*) which promotes chromosome condensation together with kinesin-5 (encoded by *Kif11*) and kinesin-13 (encoded by *Kif2*), which establish and position and the mitotic spindle, respectively [65,66,67,68].

***Centromere proteins (CENPs)*:** Centromere proteins encode components of the kinetochore complex which is the site of attachment for the mitotic spindle (reviewed in [69]). Our results reveal the profound differential regulation of *Cenp* genes in the pattern described above (Figure 5b). Of these, *Cenpf* encodes a protein shown to be critical for chromosome segregation and the orientation of the mitotic spindle.

***Aurora kinases*:** These genes encode serine/threonine kinases that promote mitosis and cell division [70]. Our results revealed the differential expression of the genes encoding aurora kinases A (*Aurka*) and B (*Aurkb*) together with the Aurka-interacting/regulatory proteins, *Aunip*, *Hmmr*, and *Tpx2* in response to sublethal PVM infection (Figure 5c).

## 4. Discussion

In this study, we examined gene expression in the lung tissues of mice that are recovering from acute sublethal infection with PVM. Consistent with our findings in mice subjected to lethal PVM infection, we show here that the administration of *L. plantarum* to the respiratory mucosa protected against weight loss and proinflammatory cytokine production in a sublethal infection model. Furthermore, we found that treatment with *L. plantarum* had no impact on virus clearance or on post-viral airway hyperresponsiveness that develops upon recovery from a sublethal PVM infection. This latter finding was somewhat surprising given the profound inhibition of both clinical symptoms as well as the inflammatory response to acute PVM infection. This finding was also unexpected given the recent human clinical data that reported an association between the abundance of *Lactobacillus sps.* in the nasopharynx with a reduced risk of developing wheeze after acute infection with RSV [71]. We speculated that ongoing airway remodeling taking place in the *Lactobacillus*-treated mice even after the resolution of acute post-viral inflammation may result in persistent airway hyper-responsiveness. Structural changes to the airways, including those involving airway smooth muscle mass and the composition of extracellular matrix components, are prominent features of chronic asthma in humans that do not necessarily correlate with the degree of inflammation [72]. Serial measurements of airway resistance over time in these mice post-infection might be needed to clarify this issue.

Sequencing performed on RNA from lung tissue of mice undergoing recovery from acute PVM infection confirmed ongoing the *L. plantarum*-mediated suppression of the acute inflammatory response that persists into the recovery phase. Our findings also revealed a dramatic induction of genes associated with mitosis and cell cycle regulation during recovery from an acute sublethal infection. These responses were largely diminished in PVM-infected mice that were treated with *L. plantarum*. However, as noted above, we recognized that these results represent the differential expression from a variety of cell types that interact with one another at a single point in time. One or more single-cell approaches (e.g., sc-RNA-seq) might be useful in unraveling one or more of these relationships. As such, we consider these findings to be a starting point for future consideration and experimental exploration.

We were particularly interested in the differential regulation of the cell division cycle gene, *Cdc20b*, together with *Plk1*, *MCIDAS*, *Espl1*, and *Pttg1* (see Figure 4c) which were all identified as up-regulated in differentiating human airway epithelial cells in a recent report by Revinski and colleagues [51]. This report also identified the critical contributions of CDC20b in centriole formation and thus the differentiation of ciliated lung epithelial cells in mouse airway epithelial cell culture. Our results suggest that *Cdc20b* and associated genes may also act in vivo to provide critical contributions to the repair of damaged lung tissue during recovery from acute respiratory virus infection. This might be further examined in a mouse model with a lung-epithelial cell-specific deletion of this gene.

These findings have also broadened the scope of our previously established understanding of the responses to *Lactobacillus sps*. and mechanisms underlying the *Lactobacillus*-mediated protection of the airways. We previously showed that while *L. plantarum* is rapidly cleared from lung tissue [73], it is most effective when administered as long as a week prior to or within 36 h after inoculation with PVM [8,9,10,11,12]. The findings shown here, in which *L. plantarum* is administered at 24 and 48 h after inoculation with a sublethal dose of PVM, are fully consistent with the previously characterized role of *L. plantarum* in lethal infection models, i.e., limiting weight loss and suppressing the acute inflammatory response to virus infection. Collectively, these results suggest that the administration of *L. plantarum* serves to prevent uncontrolled inflammation at the earliest stages of infection, thereby limiting the extent of lung tissue damage and thus the need for subsequent mitosis associated with tissue repair.

While our previous studies were primarily focused on the impact of *L. plantarum* in PVM-infected mice, here we also examined the impact of *L. plantarum* alone on gene expression. We identified 68 genes that were up-regulated and 8 genes that were down-regulated 4-fold or more in mice treated with *L. plantarum* alone; the latter group includes the cell cycle regulatory gene *Cdc20b* (7.5-fold down-regulated from control levels). In fact, we found that treatment with *L. plantarum* alone resulted in the reduced expression of nearly all of the mitosis and cell cycle genes to levels that were significantly below baseline controls (see Figure 4a–d and Figure 5a–c). In other words, these results suggest that *L. plantarum* not only protects against the negative sequelae of an acute respiratory virus infection, it may have the capacity to protect mouse lung tissue against routine inflammatory insults, thus reducing the extent of ongoing and repetitive cell and tissue replacement. The implications of this finding and its impact on the relationship linking chronic inflammation and the development of lung cancer might be further explored [74,75,76].

In summary, our results provide significant insight into gene expression that can be used to generate and explore new hypotheses focused on repair pathways during recovery from acute respiratory virus infection. Perhaps most intriguing is the fact that our findings suggest that the direct administration of *L. plantarum* to the airways may have a more substantial and direct effect on lung homeostasis than previously considered.

## Figures and Tables

**Table 1:** **Differential gene expression in lung tissue**. Results of RNA sequencing of lung tissue from control and pvm-infected mice (inoculated on day 0) both with or without *L. plantarum* (inoculated on days 1 and 2) as described in Figure 1a. See full dataset in GSE186740.

	#Up-Regulated	#Down-Regulated
	(log_2_|FC| ≥ 2.00)	(log_2_|FC| ≤ −2.00)
**+pvm vs. control**	289	19
**+pvm vs. +pvm +*Lp***	162	44
**+pvm +*Lp* vs. control**	86	4
**+*Lp* vs. control**	68	8

## Data Availability

The RNA sequencing findings generated in this work can be found online at the NCBI GEO Database file number GSE186740.

## Supplementary Materials

The following are available online at https://www.mdpi.com/article/10.3390/pathogens10121625/s1, Figure S1. Principal component analysis (PCA); Table S1. Differential expression of inflammation-associated genes, Table S2. Differential regulation of mitosis and cell cycle-associated genes.
