# Supplementary material for: Differential Expression of Mitosis and Cell Cycle Regulatory Genes during Recovery from an Acute Respiratory Virus Infection
Source: Pathogens. 2021 Dec 15;10(12):1625. doi: 10.3390/pathogens10121625 (PMC8708581; doi:10.3390/pathogens10121625)
Supplement: Supplementary file 1 [file pathogens-10-01625-s001.zip › pathogens-1487605-Supplementary Materials.pdf]

pathogens

MDPI

Supplementary Materials:

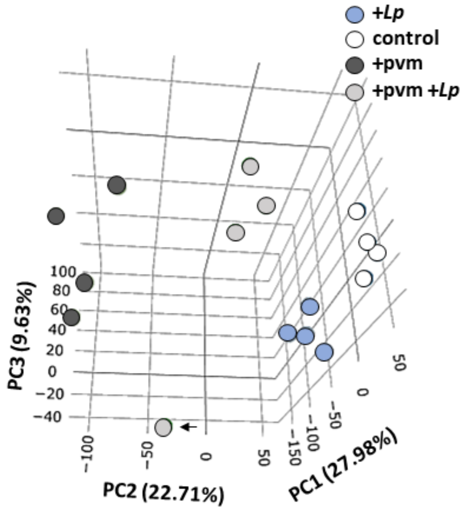

Figure S1. Principal Component Analysis (PCA). RNA was prepared from total lung tissues (n = 4 mice) on day 14 after inoculation with sublethal PVM alone (day 0), sublethal PVM (day 0) followed by *Lp* (days 1 and 2), *Lp* alone (days 1 and 2) or diluent controls at all three time points (see Fig. 1a). At the arrow, data from one mouse deviated substantially from others in the PVM + *Lp* group. This sample was omitted from further evaluation.

Table S1. Differential expression of inflammation-associated genes. Ensembl identification, gene name, and $\log_2$ fold change (FC) documenting differential regulation of proinflammatory genes from mouse lung tissue as revealed by RNA sequencing data. See also Figure 2a and GSE186740; transcripts marked with an asterisk (*) are included in Figure 2b.

	ENSEMBL ID	Gene name	Gene function	Log2|FC| +pvm *vs.* control	Log2|FC| +pvm+*Lp* vs. control	Log2|FC| +*Lp* *vs.* control
1	ENSMUSG00000037872	Ackr1	chemokine receptor	2.38608675	1.847368221	1.133091624
2	ENSMUSG00000022126	Acod1	inhibits inflammation	3.837246566	2.942333613	0.705490783
3	ENSMUSG00000028989	Angptl7	extracell matrix	2.605452368	0.500259459	0.40221042
4	ENSMUSG00000027483	Bpifa1*	antimicrobial	1.822354899	9.386988359	9.297699905
5	ENSMUSG00000027485	Bpifb1*	antimicrobial	0.860499765	4.674932218	3.500421434
6	ENSMUSG00000036887	C1qa	complement component	2.07811307	1.165540391	0.445583867
7	ENSMUSG00000036905	C1qb	complement component	2.234702123	1.199378489	0.43188276
8	ENSMUSG00000036896	C1qc	complement component	2.106360772	0.928433573	0.303162027
9	ENSMUSG00000035352	Ccl12	chemokine	2.150156111	1.437441118	0.861223811
10	ENSMUSG00000035385	Ccl2	chemokine	3.862375765	0.938916051	0.167665719
11	ENSMUSG00000026166	Ccl20	chemokine	3.378052137	3.168332839	1.643437337
12	ENSMUSG00000000982	Ccl3	chemokine	2.100903371	1.345741939	0.452897851
13	ENSMUSG00000018930	Ccl4	chemokine	2.966180339	1.858863182	0.316504949
14	ENSMUSG00000035373	Ccl7	chemokine	4.312622121	1.789014137	0.689287496
15	ENSMUSG00000009185	Ccl8	chemokine	5.087748851	2.81252292	1.014036017
16	ENSMUSG00000079227	Ccr5	chemokine receptor	2.602833603	1.374657343	0.012210853
17	ENSMUSG00000029380	Cxcl1	chemokine	2.23371154	1.424025793	-0.16747056
18	ENSMUSG00000034855	Cxcl10	chemokine	3.523902128	1.342059734	0.186105864
19	ENSMUSG00000060183	Cxcl11	chemokine	4.332361042	2.326578921	-1.872586644
20	ENSMUSG00000029379	Cxcl3	chemokine	2.93264748	1.959531072	0.309764119
21	ENSMUSG00000029417	Cxcl9	chemokine	5.515614808	3.442068107	0.681681244
22	ENSMUSG00000050232	Cxcr3	chemokine receptor	2.818190623	2.090828009	0.355501475
23	ENSMUSG00000048521	Cxcr6	chemokine receptor	2.773620263	2.211408073	0.981142859
24	ENSMUSG00000037474	Dtl	ubiquitination	2.455550787	0.296804983	-1.34702638
25	ENSMUSG00000029675	Eln	elastin	1.957561099	0.23658655	0.481993897
26	ENSMUSG00000051279	Gdf6	cytokine TGF-beta family	3.423251645	0.088292786	1.589147033
27	ENSMUSG00000074934	Grem1	inhibitor TGF-beta signaling	3.051076599	-0.094685338	-0.307934632
28	ENSMUSG00000015437	Gzmb	granzyme b	2.599964532	0.768993056	-0.71181451
29	ENSMUSG00000042385	Gzmk	granzyme k	4.325152731	2.573743915	0.471959973
30	ENSMUSG00000055170	Ifng	interferon (IFN) gamma	3.745277454	2.437222094	0.779473204
31	ENSMUSG00000020053	Igf1	insulin-like growth factor	2.080126235	0.343258981	0.430074617
32	ENSMUSG00000004296	Il12b	cytokine receptor	2.712396719	2.088522633	1.399946456
33	ENSMUSG00000000791	Il12rb1	cytokine receptor	2.187617754	1.499843543	1.04707229
34	ENSMUSG00000025746	Il6	cytokine	2.058588761	0.061158731	-0.604491064
35	ENSMUSG00000028068	Iqgap3	TGF-beta regulator	3.002263265	0.506649039	-1.723395856
36	ENSMUSG00000075502	Kbtbd6	ubiquitin ligase complex	2.23621592	0.043062089	-0.817739186
37	ENSMUSG00000030167	Klrc1	NK cell receptor	2.449360231	1.581455803	-0.472801753
38	ENSMUSG00000052736	Klrc2	NK cell receptor	2.175533407	1.423565465	-0.292268426
39	ENSMUSG00000026822	Lcn2	lipocalin2	2.112078351	1.100572741	0.522130465
40	ENSMUSG00000032496	Ltf*	lactoferrin	1.992547271	4.286560201	3.100820189
41	ENSMUSG00000025044	Msr1	macrophage scavenger receptor	2.05474814	0.843271115	0.017107359
42	ENSMUSG00000066108	Muc5b*	mucin	0.628880777	2.720909442	1.922841391
43	ENSMUSG00000041616	Nppa*	atrial natiuretic peptide	2.933443436	8.865166926	8.657805717
44	ENSMUSG00000028004	Npy2r	neuropeptide receptor	2.305706854	1.630568222	0.011538217
45	ENSMUSG00000051048	P4ha3	prolyl hydroxyase	2.254461939	-0.060027081	-0.205467194
46	ENSMUSG00000073530	Pappa2	metalloproteinase	3.06803496	0.448971657	0.607336488
47	ENSMUSG00000026285	Pdcd1	programmed cell death	3.577369144	1.862333704	1.14726533
48	ENSMUSG00000061100	Retnla	resistin-like alpha	4.404733538	0.914403916	-0.233039772
49	ENSMUSG00000040026	Saa3	serum amyloid a3	3.369017515	2.237460255	1.522796385
50	ENSMUSG00000025165	Sectm1a	amplifies inflammation	2.723137151	1.661536724	-0.999806351
51	ENSMUSG00000053318	Slamf8	inflammation	2.366014829	1.649682688	1.066977188

52	ENSMUSG00000069793	Slfn9	regulated by IFNs	2.720794472	0.32095464	-1.164594027
53	ENSMUSG00000029304	Spp1	secreted phosphoprotein	2.429382988	0.346849649	0.060155355
54	ENSMUSG00000001131	Timp1	metallopeptidase inhibitor	2.425745666	0.393727713	0.476193964
55	ENSMUSG00000028364	Tnc	tenascin c	2.900379529	0.176327893	0.363043471
56	ENSMUSG00000024401	Tnf	cytokine	2.082609908	1.394383319	0.309648235
57	ENSMUSG00000028965	Tnfrsf9	cytokine receptor	2.711512391	1.672183417	0.509129154
58	ENSMUSG00000044162	Tnip3	TNF interacting protien	2.208192544	1.00178983	0.434738637
59	ENSMUSG00000023992	Trem2	myeloid triggering receptor	2.471953775	1.591173988	0.838468719
60	ENSMUSG00000035186	Ubd	ubiquitin d	6.474484797	4.270454652	1.746076047
61	ENSMUSG00000069792	Wfdc17	inflammation	1.957159491	1.439550402	0.855167854

Table S2. Differential regulation of mitosis and cell cycle-associated genes. Ensembl identification, gene name, and log_2 fold change (FC) documenting differential regulation of mitosis and cell cycle associated genes in mouse lung tissue as revealed by RNA sequencing data. See also Figure 3 and GSE186740.

	ENSEMBL ID	Gene name	Gene function	Log_2|FC| +pvm *vs.* control	Log_2|FC| +pvm+*Lp* *vs.* control	Log_2|FC| +*Lp* *vs.* control
1	ENSMUSG00000046295	Ankle1	DNA damage and repair	2.757510083	0.106148628	-2.639285581
2	ENSMUSG00000036777	Anln	anillin - cell division	2.52704885	0.186980424	-0.930793506
3	ENSMUSG00000005470	Asf1b	histone deposition	2.162153678	0.141083381	-0.593642329
4	ENSMUSG00000033952	Aspm	spindle formation	2.920335427	0.294031249	-1.328060583
5	ENSMUSG00000078521	Aunip	aurora kinase interacting protein	2.796703029	0.344671486	-0.571822318
6	ENSMUSG00000027496	Aurka	aurora kinase - mitosis	2.847871379	0.465369717	-0.751855272
7	ENSMUSG00000020897	Aurkb	aurora kinase - mitosis	3.055105167	0.648737633	-0.635012432
8	ENSMUSG00000017146	Brca1	DNA repair	2.169839076	-0.059160807	-1.090042724
9	ENSMUSG00000034329	Brip1	Fanconi anemia - downstream of E2F	1.955432854	0.348133987	-0.431634922
10	ENSMUSG00000027379	Bub1	mitotic checkpoint kinase	2.571407848	0.386384859	-0.087755297
11	ENSMUSG00000040084	Bub1b	mitotic checkpoint	2.501660401	0.24478917	-0.827119462
12	ENSMUSG00000027793	Ccna1	cyclin	2.48465893	0.510398823	-0.738259001
13	ENSMUSG00000027715	Ccna2	cyclin	3.060652405	0.573638296	-0.991949125
14	ENSMUSG00000041431	Ccnb1	cyclin	3.275840682	0.55853865	-1.169825852
15	ENSMUSG00000002068	Ccne1	cyclin	2.836050329	0.398453269	-0.31142319
16	ENSMUSG00000028212	Ccne2	cyclin	2.861709789	0.673932995	-0.671869614
17	ENSMUSG00000072082	Ccnf	cyclin	2.590387305	0.816030066	-0.223733396
18	ENSMUSG00000031971	Ccsap	spndle protein	1.978526958	1.010224122	0.673965804
19	ENSMUSG00000006398	Cdc20	cyclin-dependent kinase	2.880592957	0.729078873	-0.357116431
20	ENSMUSG00000044201	Cdc25c	cyclin-dependent kinase	3.384591326	0.741893689	-1.045277275
21	ENSMUSG00000017499	Cdc6	cyclin-dependent kinase	2.534574794	0.419418986	-0.742613051
22	ENSMUSG00000048922	Cdca2	cyclin-dependent kinase	2.576494227	0.248810208	-1.057076299
23	ENSMUSG00000023505	Cdca3	cyclin-dependent kinase	2.535671915	0.397674141	-0.967556735
24	ENSMUSG00000024791	Cdca5	cyclin-dependent kinase	2.633830898	0.180280727	-0.686069733
25	ENSMUSG00000028873	Cdca8	cyclin-dependent kinase	2.822368964	0.321004644	-0.431651712
26	ENSMUSG00000019942	Cdk1	cyclin-dependent kinase	3.205126209	0.683856947	-0.947497257
27	ENSMUSG00000037628	Cdkn3	cyclin-dependent kinase	3.528603519	0.902368106	-0.569712401
28	ENSMUSG00000045328	Cenpe	centromere protein	3.02476082	0.231846659	-1.320297342
29	ENSMUSG00000026605	Cenpf	centromere protein	3.388305324	0.712618465	-1.305793372
30	ENSMUSG00000045273	Cenph	centromere protein	2.086444141	-0.374946176	-1.153895903
31	ENSMUSG00000031262	Cenpi	centromere protein	2.876341318	0.686026848	-0.58446703
32	ENSMUSG00000021714	Cenpk	centromere protein	1.946646818	-0.138202722	-1.171479358
33	ENSMUSG00000068101	Cenpm	centromere protein	2.614589743	0.677291412	-1.042925092
34	ENSMUSG00000031756	Cenpn	centromere protein	2.248578299	0.451916496	-0.270784933
35	ENSMUSG00000021391	Cenpp	centromere protein	2.550346623	0.618903045	-0.246985016
36	ENSMUSG00000024989	Cep55	mitotic phosphoprotein	3.146810177	0.649109846	-1.068641995
37	ENSMUSG00000032113	Chek1	checkpoint kinase	2.085059637	-0.070529518	-0.327816024
38	ENSMUSG00000037725	Ckap2	cytoskeletal protein - mitosis	3.134122142	0.480724647	-1.188122051
39	ENSMUSG00000048327	Ckap2l	cytoskeletal protein - mitosis	2.710566128	0.230010949	-1.188122051
40	ENSMUSG00000028044	Cks1b	cell cycle control	1.975599735	0.206879118	-0.102873365
41	ENSMUSG00000062248	Cks2	cell cycle control	2.071850054	0.253761975	-0.489113198
42	ENSMUSG00000042489	Clspn	cell cycle arrest	3.114828121	0.688427052	-0.891862857
43	ENSMUSG00000022021	Diaph3	spindle assembly checkpoint	2.514719659	0.212876944	-0.645942976
44	ENSMUSG00000037544	Dlgap5	kinetochore protein	3.126798967	0.650951928	-0.893040527
45	ENSMUSG00000020185	E2f7	transcription factor cell cycle	2.602169863	0.635870884	0.069719
46	ENSMUSG00000046179	E2f8	transcription factor - cell cycle	2.629866016	0.548385886	-0.751925914
47	ENSMUSG00000027699	Ect2	regulates cytokinesis	2.44773309	0.378791511	-0.821911937
48	ENSMUSG00000087060	Eldr	lnc RNA downstream EGFR	1.957572061	0.41392802	-1.697800653
49	ENSMUSG00000039055	Eme1	DNA endonuclease	2.977775757	0.867071756	-1.390067713

50	ENSMUSG00000051220	Ercc6l	spindle assembly checkpoint	2.155913664	0.436415184	-0.219630494
51	ENSMUSG00000029377	Ereg	epiregulin EGF like	3.03453152	0.083515183	-0.365641648
52	ENSMUSG00000022034	Esco2	chromosome separation	3.106603865	0.825249889	-1.067075061
53	ENSMUSG00000058290	Espl1	chromosome segregation	2.413772535	0.541365042	-0.531095697
54	ENSMUSG00000039748	Exo1	5' to 3' exonuclease activity	2.434732651	0.245479264	-0.810765274
55	ENSMUSG00000051225	Fam83a	cell proliferaton	2.468887614	1.165891941	0.651248392
56	ENSMUSG00000027654	Fam83d	mitosis	2.713410877	0.293209721	-0.929087709
57	ENSMUSG00000039187	Fanci	DNA repair	2.268441162	0.205420223	-0.700629764
58	ENSMUSG00000035455	Fignl1	repairs double strand breaks	2.384266397	0.102372286	-0.682535278
59	ENSMUSG00000001517	Foxm1	regulates expression of cell cycle genes	2.180005095	0.213079555	-0.4328892
60	ENSMUSG00000074802	Gas2l3	growth arrest	2.431549133	0.483124982	-0.157217464
61	ENSMUSG00000031821	Gins2	DNA replication	2.097604317	0.103601599	-0.711678093
62	ENSMUSG00000022385	Gtse1	G2 and S phase protein	2.803255917	0.678488253	-0.385066509
63	ENSMUSG00000022367	Has2	hyaluron synthase	2.894229624	0.336642498	-0.196427701
64	ENSMUSG00000025001	Hells	helicase	2.026851826	0.10557186	-0.600889806
65	ENSMUSG00000020330	Hmmr	HA receptor and mitosis	3.041728006	0.283618703	-1.576319526
66	ENSMUSG00000012443	Kif11	kinesin family	2.824664361	0.446713678	-0.90989579
67	ENSMUSG00000041498	Kif14	kinesin family	2.292384573	0.273139206	-0.703645925
68	ENSMUSG00000036768	Kif15	kinesin family	2.672553799	0.576895516	-0.662771597
69	ENSMUSG00000051378	Kif18b	kinesin family	3.187181503	0.706300568	-1.146079381
70	ENSMUSG00000003779	Kif20a	kinesin family	2.819736487	0.227450174	-1.167966802
71	ENSMUSG00000024795	Kif20b	kinesin family	2.108691711	0.063169649	-1.051776675
72	ENSMUSG00000030677	Kif22	kinesin family	2.934099344	0.757782701	-0.917039529
73	ENSMUSG00000032254	Kif23	kinesin family	2.260316906	0.326146915	-0.789075052
74	ENSMUSG00000028678	Kif2c	kinesin family	3.151962346	0.570142619	-0.888351483
75	ENSMUSG00000079553	Kifc1	kinesin family	2.580459433	0.430069419	-0.630344886
76	ENSMUSG00000024301	Kifc5b	kinesin family	2.019801795	0.086366839	-0.518882772
77	ENSMUSG00000027326	Knl1	kinetochore scaffold 1	2.845486908	0.487841667	-1.546258242
78	ENSMUSG00000027331	Knstrn	kinetochore	2.836844842	0.371729329	-0.856192921
79	ENSMUSG00000029414	Kntc1	kinetochore protein	2.78577922	0.074405901	-1.115445691
80	ENSMUSG00000098318	Lockd	downstream RNA of Cdk	2.245399506	0.212637613	-0.945396706
81	ENSMUSG00000026779	Mastl	microtubule interactions mitosis	2.514107275	0.080302543	-1.236819443
82	ENSMUSG00000074651	Mcidas	dna synthesis and cell cycle	1.976806041	0.050593499	-1.47113712
83	ENSMUSG00000026669	Mcm10	promotes strand annealing	2.330460064	0.231057661	-0.663640846
84	ENSMUSG00000026355	Mcm6	DNA replication	2.110757038	0.35464977	-0.328853251
85	ENSMUSG00000035683	Melk	cell cycle control	2.84139706	0.475737372	-0.841889274
86	ENSMUSG00000047534	Mis18bp1	chromosomal segregation during mitosis	2.865393838	0.133439485	-0.563395922
87	ENSMUSG00000031004	Mki67	proliferation marker	2.995540526	0.346242644	-1.229630586
88	ENSMUSG00000019992	Mtfr2	mitochondrial fission regulator	2.346352177	0.195071074	-1.215180667
89	ENSMUSG00000021485	Mxd3	DNA binding protein	3.082165235	0.687645352	-0.292099135
90	ENSMUSG00000015880	Ncapg	chromosome condensation	2.934998296	0.677929518	-1.146939675
91	ENSMUSG00000024056	Ndc80	component of kinetochore complex	2.97232721	0.636884651	-0.595314314
92	ENSMUSG00000039396	Neil3	DNA replication and repair	3.052163742	0.486574039	-1.457579771
93	ENSMUSG00000026683	Nuf2	kinetochore complex	2.894054952	0.244748368	-1.694015379
94	ENSMUSG00000027306	Nusap1	nucleolar and spindle protein	2.550657089	0.322921419	-1.051151908
95	ENSMUSG00000028587	Orc1	DNA replication prior to mitosis	2.678345528	0.242408124	0.162394874
96	ENSMUSG00000022033	Pbk	kinase activated by mitosis	2.902710963	0.560217543	-1.685408166
97	ENSMUSG00000040204	Pclaf	promotes anaphase - mitotic regulator	3.360620149	1.05829635	-0.396248083
98	ENSMUSG00000041064	Pif1	DNA helicase	3.346777348	0.514829178	-1.476663112
99	ENSMUSG00000020808	Pimreg	mitotic regulator	3.487922075	1.085502834	-1.435801181
100	ENSMUSG00000030867	Plk1	polo kinase	2.740184663	0.255740316	-0.545487357
101	ENSMUSG00000007080	Pole	DNA polymerase	2.267780754	0.27812282	-0.699255213
102	ENSMUSG00000038943	Prc1	regulator of cytokinesis	3.001048279	0.49801609	-1.260579349

103	ENSMUSG00000020493	Prr11	cell cycle	2.618163941	0.355542326	-0.859035889
104	ENSMUSG00000068744	Psrc1	mitosis	2.514150422	0.835904119	-0.381449151
105	ENSMUSG00000020415	Pttg1	sister chromatid separation	2.032744663	0.384606401	-0.300894042
106	ENSMUSG00000027323	Rad51	DNA repair double strand breaks	2.61020718	0.441686873	-0.766656277
107	ENSMUSG00000078773	Rad54b	DNA repair and recombination	2.373256248	0.558754018	-0.721271698
108	ENSMUSG00000028702	Rad54l	DNA repair and recombination	2.284357478	0.12652587	-0.113937487
109	ENSMUSG00000037991	Rmi2	homologous recomb DNA re-pair	2.367534828	0.939216344	0.09833412
110	ENSMUSG00000020649	Rrm2	ribonucleotide reductase - cell cycle	2.822460445	0.698514857	-0.816808616
111	ENSMUSG00000026955	Sapcd2	mitosis spindle orientation	2.877049852	1.10976583	-0.991685273
112	ENSMUSG00000023940	Sgo1	shugoshin complexes with cen-trom	2.852551117	0.131883586	-1.039480114
113	ENSMUSG00000026039	Sgo2a	shugoshin2a - complexes with centrom	2.500346219	0.298139451	-1.071396042
114	ENSMUSG00000036223	Ska1	spindle and kinetochore	2.793963988	0.614318219	-0.583397651
115	ENSMUSG00000021965	Ska3	spindle and kinetochore	3.277200733	1.058113875	-0.592262959
116	ENSMUSG00000002055	Spag5	mitotic spindle protein	2.961294683	0.57574938	-1.091077244
117	ENSMUSG00000074476	Spc24	kinetochore protein	2.27354983	0.326521994	-0.858659128
118	ENSMUSG00000005233	Spc25	kinetochore component	2.658101471	0.373034126	-1.171839185
119	ENSMUSG00000069910	Spdl1	mitotic checkpoint	2.433463435	0.274126862	-0.504599557
120	ENSMUSG00000028718	Stil	centriolar assembly	3.023519146	0.555106352	-1.221371642
121	ENSMUSG00000037313	Tacc3	kinetochore stabilization	2.180987998	0.362589055	-0.728907378
122	ENSMUSG00000046591	Ticrr	checkpoint regulator cell cycle progression	3.850003753	1.601724134	-0.129732876
123	ENSMUSG00000020914	Top2a	DNA topoisomerase	3.02170854	0.504335111	-1.051211495
124	ENSMUSG00000027469	Tpx2	interacts with aurora kinases	3.051288509	0.557361584	-0.571184581
125	ENSMUSG00000021569	Trip13	spindle assembly	2.546215095	0.886240801	-0.114299175
126	ENSMUSG00000038379	Ttk	kinase alignment at centromere	2.896606324	0.336296986	-1.470761585
127	ENSMUSG00000001403	Ube2c	cell cycle regulator	3.028672921	0.608055992	-0.593410706
128	ENSMUSG00000026429	Ube2t	ubiquitin conjugating enzyme	2.059757554	0.047246791	-0.009567983
129	ENSMUSG00000001228	Uhrf1	ubiquitin ligase	2.637970625	0.451316623	-0.63915275
130	ENSMUSG00000021614	Vcan	versican cell cycling	2.68672228	0.441900721	-0.206780498
131	ENSMUSG00000032400	Zwilch	kinetochore protein	1.961345463	-0.081855897	-0.826550872
